# T-cell exhaustion signatures characterize the immune landscape and predict HCC prognosis *via* integrating single-cell RNA-seq and bulk RNA-sequencing

**DOI:** 10.3389/fimmu.2023.1137025

**Published:** 2023-03-15

**Authors:** Hao Chi, Songyun Zhao, Jinyan Yang, Xinrui Gao, Gaoge Peng, Jinhao Zhang, Xixi Xie, Guobin Song, Ke Xu, Zhijia Xia, Shi Chen, Jinqiu Zhao

**Affiliations:** ^1^ Clinical Medical College, Southwest Medical University, Luzhou, China; ^2^ Department of Neurosurgery, Wuxi People’s Hospital Affiliated to Nanjing Medical University, Wuxi, China; ^3^ School of Stomatology, Southwest Medical University, Luzhou, China; ^4^ Department of Oncology, Chongqing General Hospital, Chongqing, China; ^5^ Department of General, Visceral, and Transplant Surgery, Ludwig-Maximilians-University Munich, Munich, Germany; ^6^ Clinical Molecular Medicine Testing Center, The First Affiliated Hospital of Chongqing Medical University, Chongqing, China; ^7^ Department of Infectious Diseases, The First Affiliated Hospital of Chongqing Medical University, Chongqing, China

**Keywords:** T-cell exhaustion, HCC, single-cell RNA-seq, machine learning, tumor microenvironment, immunotherapy, predictive signature

## Abstract

**Background:**

Hepatocellular carcinoma (HCC), the third most prevalent cause of cancer-related death, is a frequent primary liver cancer with a high rate of morbidity and mortality. T-cell depletion (TEX) is a progressive decline in T-cell function due to continuous stimulation of the TCR in the presence of sustained antigen exposure. Numerous studies have shown that TEX plays an essential role in the antitumor immune process and is significantly associated with patient prognosis. Hence, it is important to gain insight into the potential role of T cell depletion in the tumor microenvironment. The purpose of this study was to develop a trustworthy TEX-based signature using single-cell RNA-seq (scRNA-seq) and high-throughput RNA sequencing, opening up new avenues for evaluating the prognosis and immunotherapeutic response of HCC patients.

**Methods:**

The International Cancer Genome Consortium (ICGC) and The Cancer Genome Atlas (TCGA) databases were used to download RNA-seq information for HCC patients. The 10x scRNA-seq. data of HCC were downloaded from GSE166635, and UMAP was used for clustering descending, and subgroup identification. TEX-related genes were identified by gene set variance analysis (GSVA) and weighted gene correlation network analysis (WGCNA). Afterward, we established a prognostic TEX signature using LASSO-Cox analysis. External validation was performed in the ICGC cohort. Immunotherapy response was assessed by the IMvigor210, GSE78220, GSE79671, and GSE91061cohorts. In addition, differences in mutational landscape and chemotherapy sensitivity between different risk groups were investigated. Finally, the differential expression of TEX genes was verified by qRT-PCR.

**Result:**

11 TEX genes were thought to be highly predictive of the prognosis of HCC and substantially related to HCC prognosis. Patients in the low-risk group had a greater overall survival rate than those in the high-risk group, according to multivariate analysis, which also revealed that the model was an independent predictor of HCC. The predictive efficacy of columnar maps created from clinical features and risk scores was strong.

**Conclusion:**

TEX signature and column line plots showed good predictive performance, providing a new perspective for assessing pre-immune efficacy, which will be useful for future precision immuno-oncology studies.

## 1 Introduction

The most prevalent kind of cancer globally and the main cause of cancer mortality in China is primary liver cancer (1, 2). Approximately 75% to 85% of patients with initial liver cancer are hepatocellular carcinoma (LIHC) (3). Since the initial symptoms of HCC patients are not obvious, most of them are clinically detected at a late stage, however, cancer has already spread and the cure rate is very low at this time (4). Despite important advances in the treatment of LIHC, such as PD-1/PD-L1 inhibitors, the prognosis for patients with advanced LIHC remains poor (5) due to its metastatic and recurrent. The extreme heterogeneity exhibited by different individuals and different sites of LIHC urgently requires us to find new and reliable biomarkers.

Usually, after the body is infected by a pathogen, the initial T cells are activated by antigen, co-stimulation, and inflammation and proliferate exponentially towards effector T cells and memory T cells (6). However, in patients with cancer, T cells are continuously stimulated by prolonged exposure to persistent antigens and inflammation, and the inactive T cells gradually lose their effector functions and begin to lose their memory T cell characteristics, a process known as T cell Exhaustion (6–8). T cell depletion is considered to be one of the major factors of immune dysfunction in cancer patients. Several recent studies have found that blocking co-inhibitory receptors on the surface of depleted CD8+ T cells (CD8+Tex), such as programmed death receptor 1 (PD-1), reactivates the cytolytic effect of T cells (9, 10). The advent of ICB has helped us to establish a new paradigm for cancer treatment that has produced durable responses in a limited patient population. Despite the early success of ICB, the mechanism of action behind ICB and TEX still requires further study.

In recent years, the important role of the tumor immune microenvironment (TIME) in cancer progression and treatment response has emphasized the importance of identifying tumor immune profiles and immune characteristics of patients with different tumors (11). Tumor-infiltrating T cells constitute an important component of TIME and play a key role in recognizing and killing tumor cells. However, due to the level and number of expressed inhibitory receptors (IRS), most infiltrating T cells become “depleted”, leading to cancer immune evasion (12). Depleted T cells exhibit a distinct epigenetic profile, which may lead to adverse responses to immunotherapy (13).

With the rapid development of high-throughput sequencing and but single-cell sequencing, a large number of methods are being used to define biomarkers of disease, and notable achievements have been made in the prognosis prediction of cancer (14–23). More evidence suggests that the onset of TEX is a gradual and dynamic process (24). Hence, this study aimed to identify and characterize patients with different TEX profiles. In order to identify TEX-related genes with high prognostic value, we combined bulk sequencing (bulk-seq) and single-cell RNA sequencing (scRNA-seq) data from HCC samples. This study built prognostic features based on multiple TEX-related genes using a variety of analytical techniques in an effort to clarify the connection between TEX process-related genes and the prognosis and progression of HCC. The goal of this study was to investigate the effect of TEX-related genes on the prognosis of HCC.

## 2 Materials and methods

### 2.1 Source of raw data

Two TIHC samples were downloaded from the GSE166635 series with 10× scRNA-seq data, the two samples included 15941 cells and 8696 cells, respectively. The transcriptome data, somatic mutation, and clinical materials of the normal sample (n=50) and HCC sample (n=374) were downloaded from TCGA (https://portal.gdc.cancer.gov/). RNA-seq data and clinical information for 231 tumor samples were obtained from the ICGC database (https://dcc.icgc.org/projects/LIRI-JP). IMvigor210 (http://research-pub.gene.com/IMvigor210CoreBiologies/), GSE78220, GSE79671, and GSE91061 (https://www.ncbi.nlm.nih.gov/geo/) for the assessment and prediction of the extent of TEX signature response to tumor immunotherapy.

### 2.2 Data processing of 10×scRNA-Seq

We processed single-cell sequencing data of HCC by the following methods. First, we use the “Seurat” R package to convert 10× scRNA-seq data into Seurat objects and exclude substandard quality cells and perform quality control (QC) by calculating the percentage of mitochondrial or ribosomal genes (25). The top 2000 highly variable genes were identified using the “FindVariableFeatures” program, and 2000 additional genes were employed for descending and cell subpopulation identification using principal component analysis (PCA) and uniform manifold approximation and projection (UMAP). In order to find marker genes in various clusters, the “Find All Markers” tool was used with |Log2FC| and min. pct cutoff values set to 0.3 and 0.25, respectively. The R package “SingleR” is used for the annotation of different cell types (26). In addition, we used the “analyze_sc_clusters” function of the R package “ReactomeGSA” (27) for enrichment analysis and the “pathways” function to extract the results from different cells. Finally, the R packages “CellChat” (28) and “patchwork” were used for intercellular communication analysis and network visualization.

### 2.3 Recognition of important co-expression modules

A systems biology method for identifying genetic relationship patterns between samples is weighted correlation network analysis (WGCNA), often referred to as WGCNA. WGCNA may be used to find highly synergistic genomes and to find potential biomarker genes or therapeutic targets based on the endogeneity of the genome and the link between the genome and phenotype (18).

### 2.4 Scoring of TEX pathway activity enrichment

We refer enced a study on TEX (29), the TEX signaling pathway signaling and marker genome from the Molecular Signaling Database (MSigDB, V7.2), to estimate the TEX pathway activity score in each patient using the “GSVA” R package (16)(**Supplementary Table 1**). ImmuCellAI (http://bioinfo.life.hust.edu.cn/) is an online site that can be used for the assessment of immune cell infiltration during immunotherapy (15). ImmuCellAI-based immune cell scores were used for tumor immune checkpoint inhibitor treatment efficacy prediction using support vector machine algorithms.

### 2.5 Construction and verification of TEX signature

The optimal results were obtained by LASSO regression analysis of the training group data using the R package “glmnet”. Multivariate regression Cox analysis was performed to obtain Eleven TEXs and correlation coefficients. Then, we calculated each patient’s risk score. The calculation formula is as follows: Risk score = Expression_mRNA1_ × Coef_mRNA1_ + Expression_mRNA2_ × Coef_mRNA2_ +… Expression_mRNAn_ × Coef_mRNAn_. Based on the median value of the risk score, patients in the training group were divided into high- and low-risk groups. Kaplan–Meier survival analysis was performed, and a receiver operating characteristic curve (ROC) was constructed. To verify the predictive ability of the model, we evaluated its prognosis, sensitivity, and specificity in the testing group. Then, we verified it in the ICGC cohort according to the formula of risk score.

### 2.6 Independent prognostic analysis and nomogram construction

To determine if the TEX signature may serve as a standalone predictive factor in patients with HCC, we conducted univariate and multivariate Cox regression analysis. A nomogram for predicting OS at 1, 2, and 3 years in clinical patients was created using the “rms” R package based on the patient’s age, grade, gender, stage, T stage, and risk scores. The calibration study findings further demonstrated the precision of the nomogram prediction outcomes.

### 2.7 Functional enrichment analysis

The GO and KEGG pathways were analyzed using the “ClusterProfiler” R package. “circlize” R package visualizes the GO and KEGG results. Analysis was performed by GSVA algorithm using “c2.cp.kegg.v7.4.symbols.gmt” in MSigDB to get the differences in enrichment pathways between different risk groups.

### 2.8 Somatic mutation analysis

We utilized maftools to evaluate somatic variant data from HCC samples that were saved in mutation annotation format (MAF) (14). We calculated the tumor mutation burden (TMB) score for each HCC patient and explored the relationship between the risk score and TMB. The TMB score was calculated as follows: (total mutations/total covered bases) × 10^6^ (24). The prognostic value of TMB in HCC was investigated using Kaplan-Meier analysis in the R package.

### 2.9 Correlation analysis of TEX signature and immune microenvironment

Correlations between risk scores and tumor-infiltrating immune cells were assessed using seven algorithms, including XCELL, TIMER, QUANTISEQ, MCPCOUNTER, EPIC, CIBERSORT-ABS, and CIBERSORT. Using 20 molecules of suppressive immune checkpoints from Auslander’s study, we evaluated the expression levels of immune checkpoints between the high- and low-risk groups. In addition, TME scores, including stromal score, immune score, and estimated score, were calculated for both groups using the R package “ESTIMATE”.

### 2.10 Immunotherapy prediction and chemotherapy sensitivity analysis

We collected three GEO immunotherapy cohorts and the IMvigor210 cohort to investigate the correlation between the TEX signature and immunotherapy. We processed the data using the “IMvigor210CoreBiologies” R package in the IMvigor210 cohort. Based on the public pharmacological Web portal, Genomics of Drug Sensitivity in Cancer (GDSC) (https://www.cancerrxgene.org/), we estimated the half-maximal inhibitory concentration (IC50) of common chemotherapeutic drugs for HCC by “pRRophetic” R package.

### 2.11 Cell lines

All cells were cultured in a 37°C incubator in an atmosphere of 5% CO2. The normal human hepatocyte cell line HL-7702, human live cancer cell line Huh7 and human liver cancer cell line Hep3B were from the Chinese Academy of Sciences (Shanghai, China). Cell culture medium, plates, and dishes were from Thermo Fisher Scientific (Invitrogen, USA) and Corning Inc. (NY, USA). HL-7702, Huh7, and Hep3B cultured in Dulbecco’s modified Eagle medium supplemented with 10% fetal bovine serum and 10,000 U/mL of penicillin-streptomycin.

### 2.12 RNA extraction and qRT-PCR

HL-7702 cells, Huh7 cells, and Hep3B cells were detached and seeded into 60 mm dishes at the initial density of 1×106 cells/well overnight. Subsequently, total RNA was extracted using RNA Eazy Fast Tissue/Cell Kit (TIANGEN Biotech. Co., Bejing). The quality of RNA was measured using a NanoDrop 2000 Spectrophotometer (Thermo Fisher Scientific Inc., USA). Total RNA (2 μg)) was reverse transcribed into cDNA with the FastKing RT Kit (TIANGEN Biotech. Co., Bejing). We performed real-time PCR using the SuperReal PreMix Plus (TIANGEN Biotech. Co., Bejing) and a steponeplus real-time PCR system (Applied Biosystems) according to the manufacturer’s instructions. The relative expression levels in terms of fold changes of target genes were calculated by the 2^-△△CT^ method. The sequences of the primers are shown in **Supplementary Table 2**.

### 2.13 Statistical analysis

R software version 4.1.3 was used to conduct the statistical analysis, and p-values and FDR (false discovery rate) q-values below 0.05 were regarded as statistically significant.

## 3 Results

### 3.1 ScRNA-Seq analysis of HCC samples

**Figure 1:**
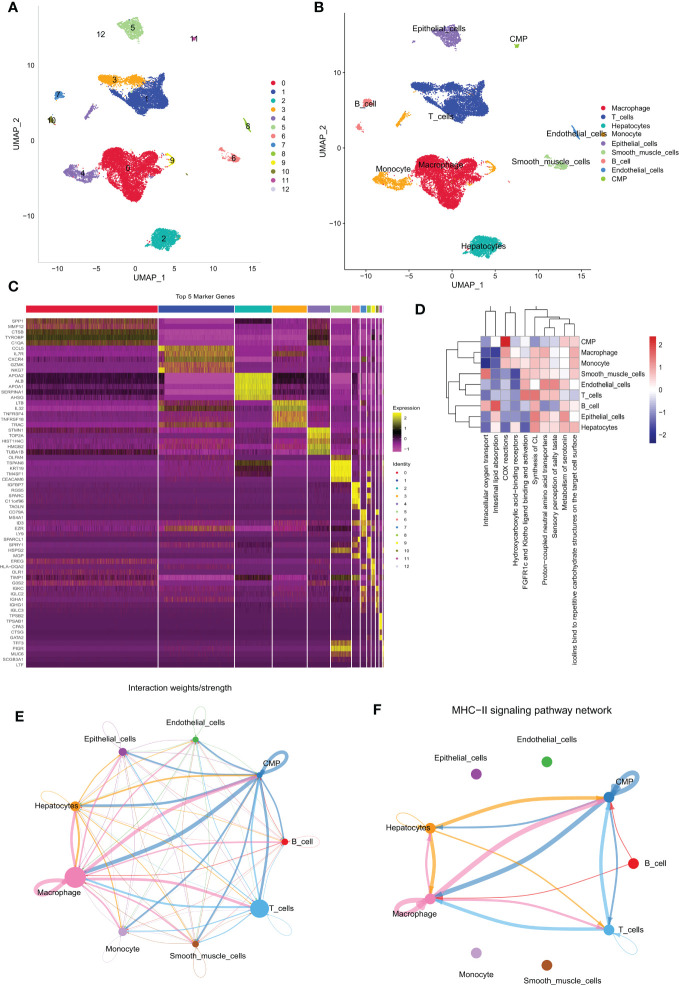
Different cell clustering in 10x scRNA-seq data of hepatocellular carcinoma and further analysis. **(A, B)** Cluster annotation and cell type identification by means of UMAP. **(C)** Heat map of marker genes for different cell types. **(D)** Functional enrichment analysis of all cell types using the “ReactomeGSA” package. **(E, F)** Cellular communication networks were inferred by calculating the likelihood of communication. Intercellular communication network studies show that HLA-DPA 1-CD 4 plays an important role in the intercellular communication network. scRNA-seq, single cell RNA sequencing; UMAP, Unified Flowform Approximation and Projection.

The primary design of this study can be known from the graphical flow chart (**Supplementary Figure 1**). We downloaded 10x scRNA-seq data from the GSE166635 dataset for two LIHC samples. The first two samples of QC contained 15941 and 8696 cells, respectively, and the second two samples of QC identified 13064 and 5922 cells (**Supplementary Figure 2A**). We showed the first 2000 highly variable genes in (**Supplementary Figure 2B**). A total of 13 distinct cell subgroups were identified after descending clustering using UMAP analysis (**Figure 1A**). The SingleR package was then used to annotate and visualize the clustering of the downscaled cell types. Overall, we identified nine major cell types in this step, including monocytes, macrophages, myeloid progenitor cells, hepatocytes, endothelial cells, smooth muscle cells, epithelial cells, B cells, and T cells (**Figure 1B**). **Figure 1C** illustrates marker gene expression in these cell subpopulations. ReactomeGSA functional enrichment analysis showed that T cells are mainly involved in the Synthesis of CL and FGFR1c and Klotho ligand binding and activation-related pathways (**Figure 1D**). We investigated the cell-cell communication network by calculating communication probability (**Figure 1E**, **Supplementary Figure 3A**). In addition, we inferred cell-cell communication networks based on specific pathways and ligand receptors. We found that the MHC-II signaling pathway plays a crucial role in the communication network concerning T cells (**Figure 1F**, **Supplementary Figure 3B**). The immune system recognizes tumor cell complexity to a large extent through major histocompatibility complexes (MHCs). High expression of MHC-II in tumors is essential for antigen presentation by T lymphocytes, and the role of CD4+ T lymphocytes in antitumor immunity is increasingly appreciated (25).

### 3.2 Identification of candidate TEX-related genes

**Figure 2:**
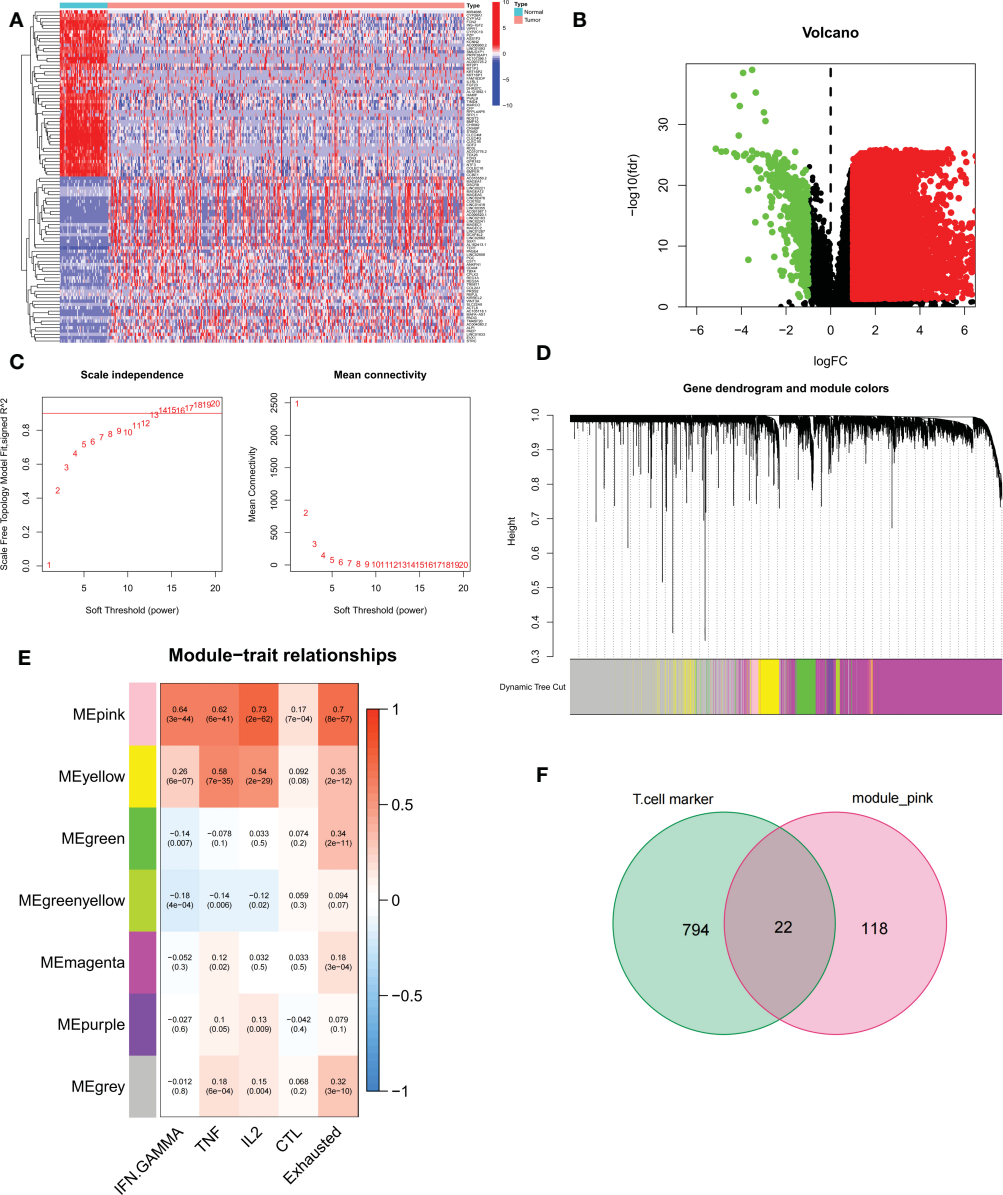
Identification of candidate T cell exhaustion-related genes. **(A, B)** Heat map and volcano map of differentially expressed genes in the TCGA cohort. **(C)** Scale independence and average connectivity. **(D)** Cluster dendrogram. **(E)** Heatmap of the correlation between TEX pathway and exhausted T cell scores and modules. **(F)** Venn diagram of T-cell marker genes and pink modules.

We first performed differential expression analysis of the TCGA-LIHC cohort using the limma package and found a total of 14,106 DEGs. In liver cancer tissues, the vast majority of genes were upregulated in expression (**Figure 2B**). The 50 up-regulated genes and 50 down-regulated genes with the largest differential changes were shown on the heat map (**Figure 2A**). Next, using GSVA, we obtained enrichment scores for the four pathways associated with TEX for each sample, and through the ImmuCellAI online website, we obtained enrichment scores for depleted T cells directly. Using these DEGs, we identified the key modules in the TCGA cohort most associated with the progression of T cell depletion. During the construction of the co-expression network, we observed a soft threshold power β of 13 when the scale-free topology fit index reached 0.9 (**Figure 2C**). We then used the “merged dynamics” algorithm to obtain seven modules (**Figure 2D**). Based on the correlation coefficient and P-value, we found that the pink module had the best correlation with the score associated with TEX progression (**Figure 2E**) (*P*<0.001), so the pink module was selected as the key module. We selected the intersection of T-cell marker genes and pink module genes and finally obtained 22 candidate TEX-associated genes (**Figure 2F**).

### 3.3 TEX signature establishment and external validation

**Figure 3:**
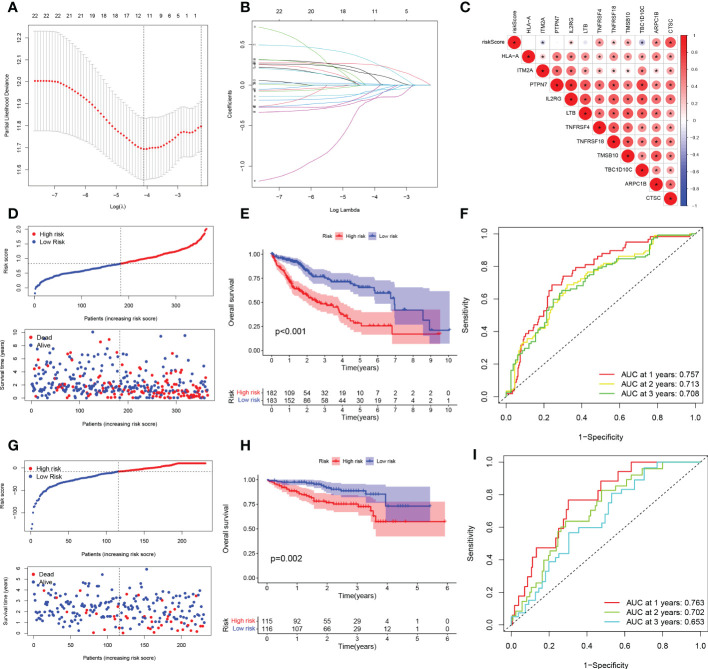
TEX signature establishment and external validation. **(A)** Lasso regression profiles of TEXs to avoid over-fitting. **(B)** 10-fold cross-validation of variable selection with Lasso. **(C)** Correlation of risk scores and 11-TEX genes. **(D, G)** Distribution of risk scores and patient survival between low and high risk groups in the TCGA cohort and the ICGC cohort. **(E, H)** KM curve compares the overall HCC patients between LR and HR groups in the TCGA cohort and the IGCG cohort. **(F, I)** Time-dependent ROC curves analysis in the TCGA cohort and the ICGC cohort.

To exclude co-expressed TEX genes and avoid over-fitting, we constructed a predictive prognostic model consisting of TEX genes by lasso regression analysis. They were HLA-A, ITM2A, PTPN7, IL2RG, LTB, TNFRSF4, TNFRSF18, TMSB10, TBC1D10C, ARPC1B, and CTSC (**Figures 3A, B**). A linear prediction model was developed based on the weighted regression coefficients of 11 prognosis-related TEXs, calculated as risk score = (-0.0712 x HLA-A exp) + (-0.0878 x ITM2A exp) + (-0.1846 x PTPN7 exp) + (0.1181 x IL2RG exp) + (-0.1403 x LTB exp) + (0.2926 x TNFRSF4 exp) + (-0.0811 x TNFRSF18 exp) + (0.0145 x TMSB10 exp) + (-0.1721 x TBC1D10C exp) + (0.1368 x ARPC1B exp) + (0.2523 x CTSC exp). Of these, CTSC, ARPC1B, TMSB10, TNFRSF18, TNFRSF4, and IL2RG showed significant positive correlations with risk scores, with CTSC showing the strongest positive correlation. In addition, TBC1D10C and ITM2A showed a significant negative correlation with the risk coefficient (**Figure 3C**). To demonstrate the stability and reliable generalization of our model, the TCGA-LIHC cohort was used as the internal training set, and the ICGC-LIHC cohort as the external validation cohort. Risk scores were calculated separately for each sample in the TCGA training cohort and the ICGC validation cohort based on the same risk formula, and we could find that when the risk of LIHC patients was elevated in both cohorts, patients exhibited a survival disadvantage of reduced OS and increased mortality (**Figures 3D, G**). Based on the median risk score, we could divide the patients into two subgroups of HR and LR to explore the prognostic differences between the HR and LR groups. The Kaplan-Meier curves showed a significant difference in prognosis between the HR and LR patients in these two cohorts, respectively, with a more significant survival advantage for patients in the LR group (**Figures 3E, H**). The ROC curve was used as a tool to predict the survival time of patients at 1-, 2-, and 3- years, with AUCs of 0.757, 0.713, and 0.708 for the TCGA-LIHC cohort, respectively. The AUCs for the ICGC-LIHC cohort was 0.763, 0.702, and 0.653, respectively (**Figures 3F, I**). This indicates that the model has an excellent predictive effect.

### 3.4 Creation of nomograms based on TEX signatures combined with clinical characteristics

**Figure 4:**
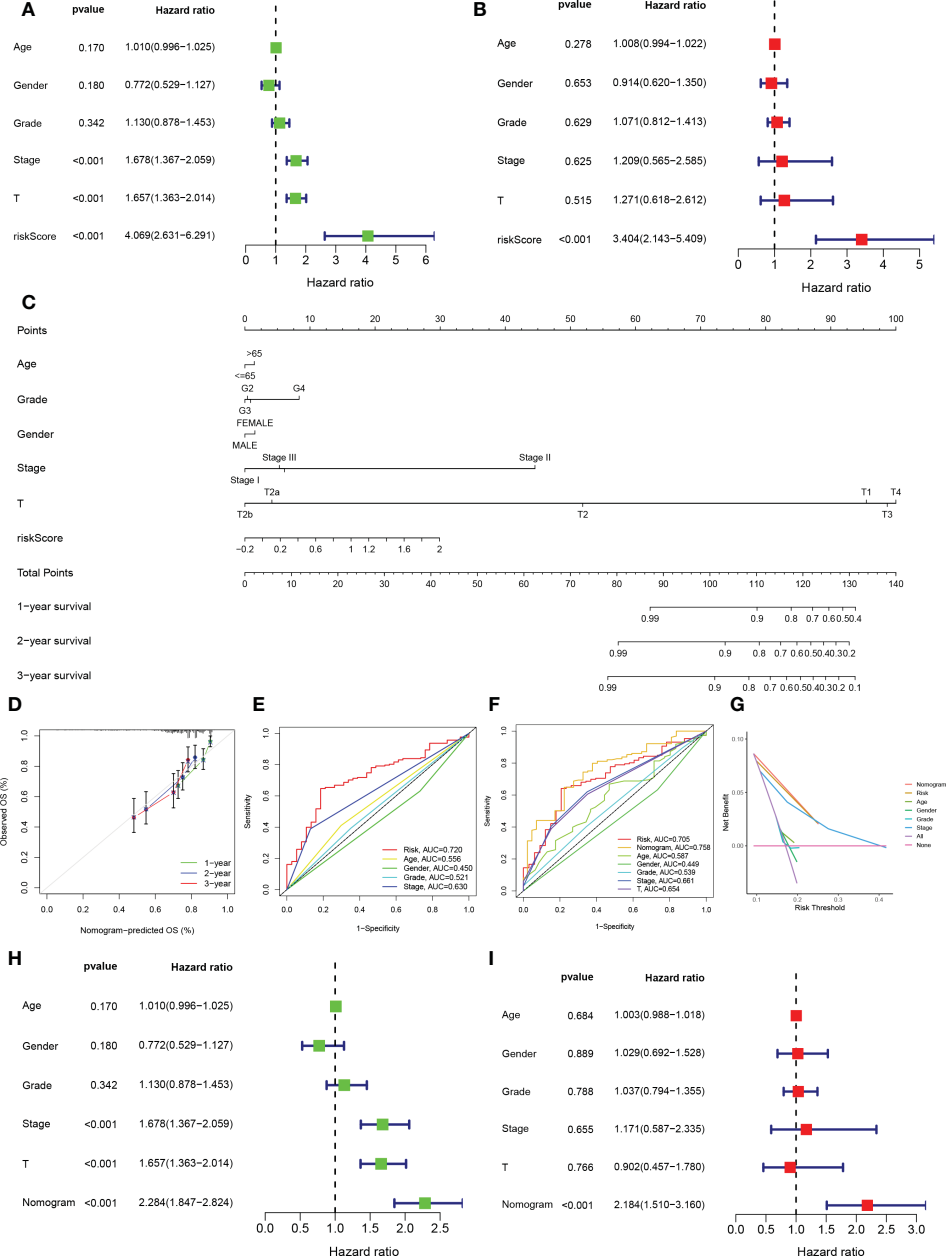
Creation of nomograms based on TEX signature combined with clinical characteristics. **(A)** Univariate and **(B)** multivariate COX regression analysis of the signature and different clinical features. **(C)** A Nomogram combining the age, grade, gender, stage, T stage, and risk score. **(D)** The calibration curve of the constructed Nomogram of 1-year, 2-year, and 3-year survival. **(E)** Time-dependent ROC curves analysis. **(F)** The Nomogram’s time-dependent ROC curves. **(G)** Decision curve analysis. **(H)** Univariate and **(I)** multivariate COX regression analysis of the Nomogram and different clinical features.

To validate the reliability and clinical value of the biological signature constructed based on TEX as a predictor of prognosis, we included a comparison of each HCC patient’s risk score with both common clinical indicators and observed the correlation of each factor with patient prognosis after successive univariate and multivariate Cox analyses. Based on the analysis of the results, it is clear that in the univariate cox analysis, Stage, T-stage, and riskscore (*P*<0.001) were all prognostic factors significantly associated with patient prognosis (**Figure 4A**). However, after multifactorial cox analysis, only the risk score (*P*<0.001) was significant (**Figure 4B**). Based on the above analysis, in order to be able to predict patients’ prognosis quantitatively and to inform clinical decision-making. We integrated the risk scores and their clinical indicators to construct Nomogram plots as a means of predicting the probability of prognostic survival at 1, 2, and 3 years (**Figure 4C**). Calibration analysis showed that the prediction curves for OS for patients at 1, 2, and 3 years were highly similar to the ideal 45-degree calibration line, indicating excellent stability of the Nomogram plot (**Figure 4D**). We then compared the Nomogram, risk, and common clinicopathological features, where in **Figure 4E** risk (AUC=0.720) has a much greater AUC value than the rest of the pathological features, and we then included the Nomogram in the comparison. The results showed that both risk (AUC=0.705) and Nomogram (AUC=0.758) had more accurate predictive performance and discriminatory power than a single independent clinical indicator (**Figure 4F**). Subsequently, DCA showed that Nomogram and risk yielded greater net benefit and predictive benefit, indicating that both the model’s risk score and Nomogram could be used as primary decision factors (**Figure 4G**). In addition, to further validate that the nomogram is a reliable tool for predicting patient prognosis, we supplemented this with univariate and multifactor cox analyses of Nomogram versus clinical indicators, which showed that the p-value for Nomogram was less than 0.001 in both univariate and multifactor cox analyses (**Figures 4H, I**). Combined with these results, this suggests that our TEX signature is more practical and influential for clinical decision-making and is more suitable as a clinical decision tool for predicting the prognosis of patients with HCC in the clinical setting.

### 3.5 Clinical correlation and survival analysis of TEX in patients with HCC

**Figure 5:**
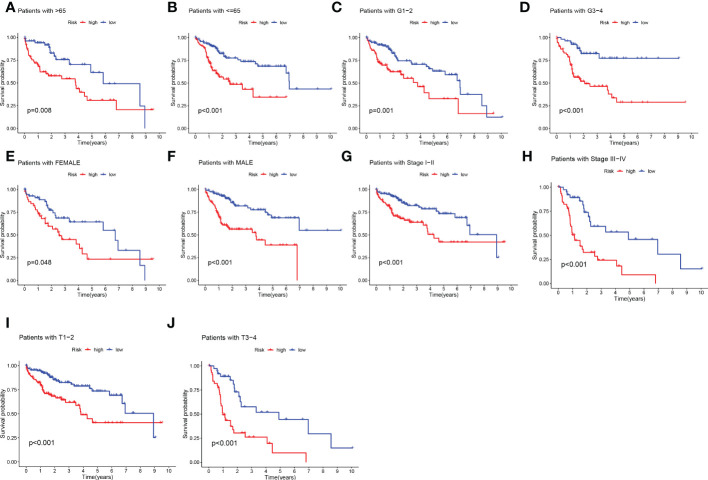
Clinical correlation and survival analysis of TEX genes in patients with HCC. **(A, B)** age, **(C, D)** pathological grade, **(E, F)** gender, **(G, H)** pathological stage, **(I, J)**, and T stage.

Given the significant differences in OS between HR groups and LR groups in individual clinical characteristics, in order to more specifically explore and compare such differences, we divided LIHC patients into five different subgroups based on clinical characteristics. These were age (≤65 and >65 years), pathological stage (I-II and III-IV), gender (female and male), pathological grade (G1-2 and G3-4), and T stage (T1-2 and T3-4). Notably, in all subgroups, LR patients had a significant survival advantage in terms of longer survival time compared to HR patients (**Figures 5A-J**). Based on the analysis of the results, we are even more convinced that the TEX signature was a reliable clinical prediction tool.

### 3.6 Distribution of patients in the HR group and LR group in different clinical subtypes

**Figure 6:**
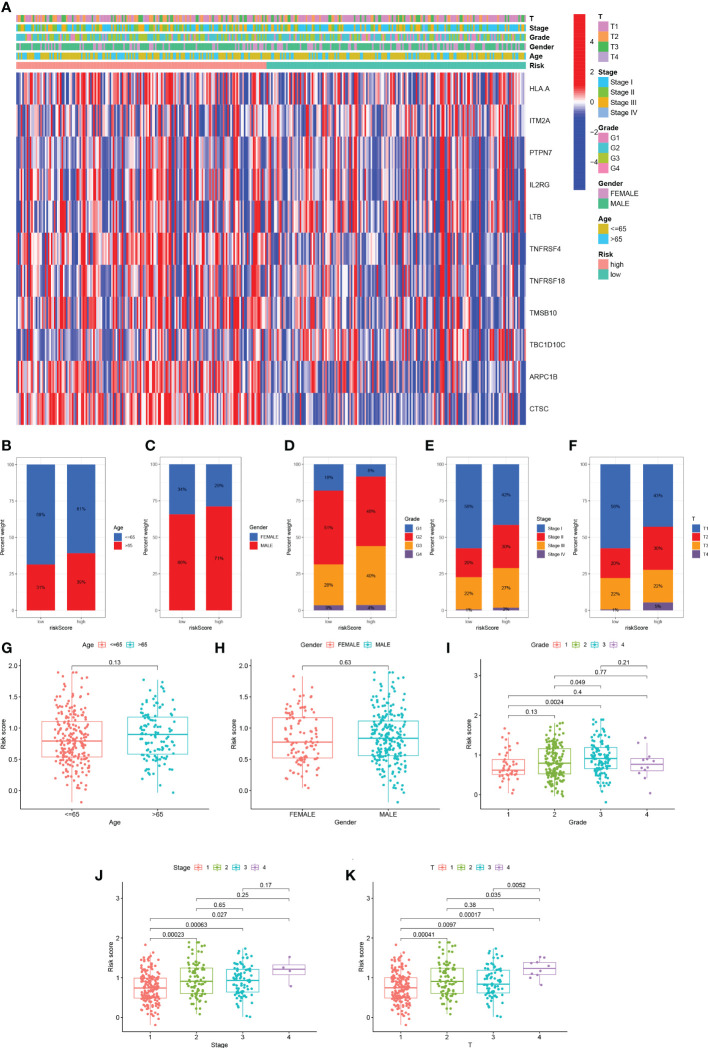
Distribution of risk scores in different clinical subtypes. **(A)** Heatmap of clinicopathological variables in HR group and LR group. **(B-F)** The proportion of patients with different clinical subtypes (Age, Gender, Grade, Stage, T stage) in the HR group and LR group. **(G-K)** Risk score distribution of different clinical subtypes.

We analyzed the expression of 11 TEX genes in the HR and LR groups and the distribution of different clinical subtypes (**Figure 6A**). Then we counted the proportion of patients with different clinical subtypes in the HR group and LR group and expressed it by the bar chart (**Figures 6B-F**). Among them, patients in the ≥ 65 years old, grade 3, stage II, and T2 accounted for a greater proportion of patients in the HR group. Risk score analysis was also performed on HCC patients by age, gender, grade, stage, and T stage to reveal the relationship between risk scores and prognosis in clinicopathological variables (**Figures 6G-K**). The results showed that there were significant differences in risk scores among patients with different grades, stages, and T stage, and patients with higher stage had higher risk scores. Therefore, we concluded that there was a significant positive correlation between risk scores and clinicopathological variables.

### 3.7 Function enrichment analysis

**Figure 7:**
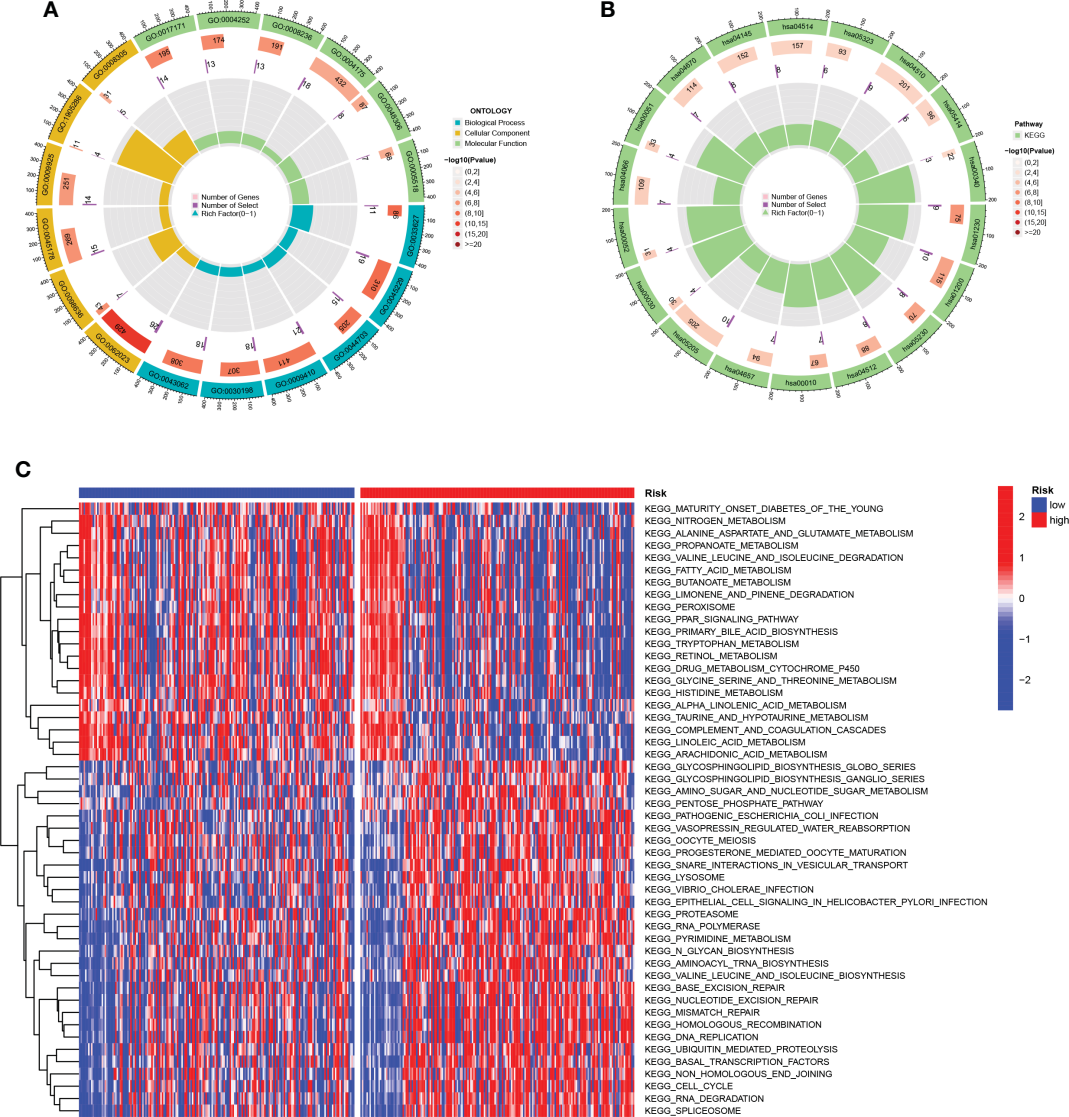
Function enrichment analysis. **(A)** GO enrichment pathway. **(B)** KEGG enrichment pathways. **(C)** Heatmap of differentially enriched pathways between the HR group and LR group.

The results of GO analysis can be divided into three categories: biological process, cellular component, and molecular function. Where in biological processes, such as cell adhesion mediated by integrin, external encapsulating structure organization, multi-organism reproductive process; Cellular components, such as serine-type peptidase complex and protein complex involved in cell adhesion; And molecular functions, Such as calcium-dependent protein binding, endopeptidase activity, monosaccharide binding and serine-type peptidase Pathways such as activity were significantly enriched (**Figure 7A**). KEGG pathways were enriched in ECM-receptor interaction, IL-17 signaling pathway, HIF-1 signaling pathway, and Leukocyte transendothelial Pathways such as migration, Phagosome, Cell adhesion molecules, and metabolism of Rheumatoid arthritis and cancer substances (**Figure 7B**). For the HR group and LR group, the differentially enriched KEGG pathways between the two groups were analyzed by GSVA (**Figure 7C**). Mature-onset diabetes of the young, peroxisome, peroxisome proliferators-activated receptors signaling pathway and drug metabolism cytochrome p450 were the pathways that were substantially enriched in 11 TEXRGs in the low-risk group. RNA polymerase, ubiquitin-mediated proteolysis in the high-risk group, ganglio series, vibrio cholerae infection, lysosome, and cell growth and division were enriched.

### 3.8 TMB analysis and survival analysis of TMB

**Figure 8:**
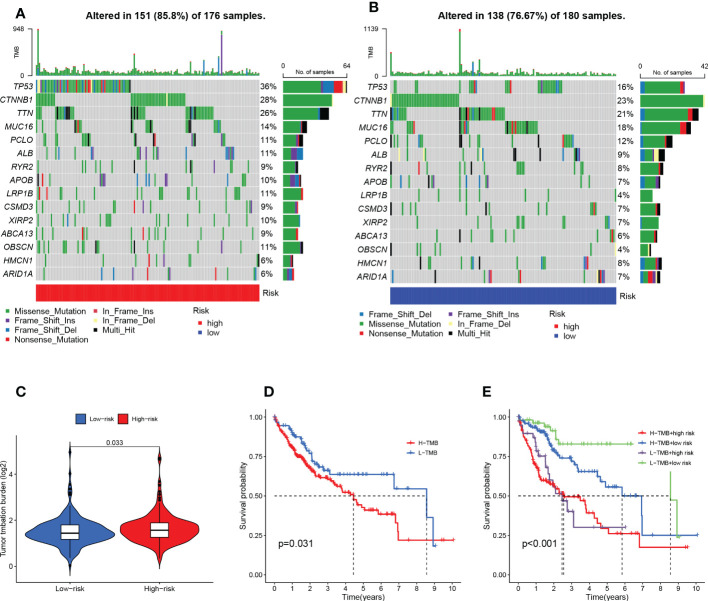
TMB analysis and survival analysis of TMB and risk scores. **(A)** Mutation analysis of HR group **(B)** Mutation analysis of LR group. **(C)** Violin plot revealing the distinction between HR and LR groups in TMB. **(D)** Kaplan-Meier curves for the high- and low-TMB groups. **(E)** Kaplan-Meier curves for the four groups divided by risk score and TMB.

It is well known that genetic mutations are a condition for tumorigenesis. In the TCGA database, we visualized and correlated somatic mutation data based on the TEX signature in combination with HR and LR groups. The three genes with the highest mutation frequencies in the HR group were TP53 (36%), CTNNB1 (28%), and TTN (26%), while the three genes with the highest mutation frequencies in the LR group were CTNNB1 (23%), TTN (21%), and MUC16 (18%) (**Figures 8A, B**). Different mutation statuses and expression patterns in the wild type have been demonstrated to produce various clinical consequences in the immune response (26, 30). TMB analysis of the HR group and LR group showed a significant difference between the two (*P*=0.033), with higher TMB in the HR group (**Figure 8C**). KM analysis was performed based on the median of the obtained TMB values divided into high- and low-TMB groups and further revealed that the low-TMB group had a better prognosis (*P*=0.031), suggesting that TMB may be an indicator of poor prognosis in patients with HCC (**Figure 8D**). The joint application of the risk scores and TMB was used to classify patients into four subgroups for survival assessment, and the low-TMB and low-risk group had the best prognosis (*P*<0.001), demonstrating the validity of the model and screening for the best prognostic subgroups for clinical use (**Figure 8E**).

### 3.9 TEX risk score predicts tumor microenvironment and immune cell infiltration

**Figure 9:**
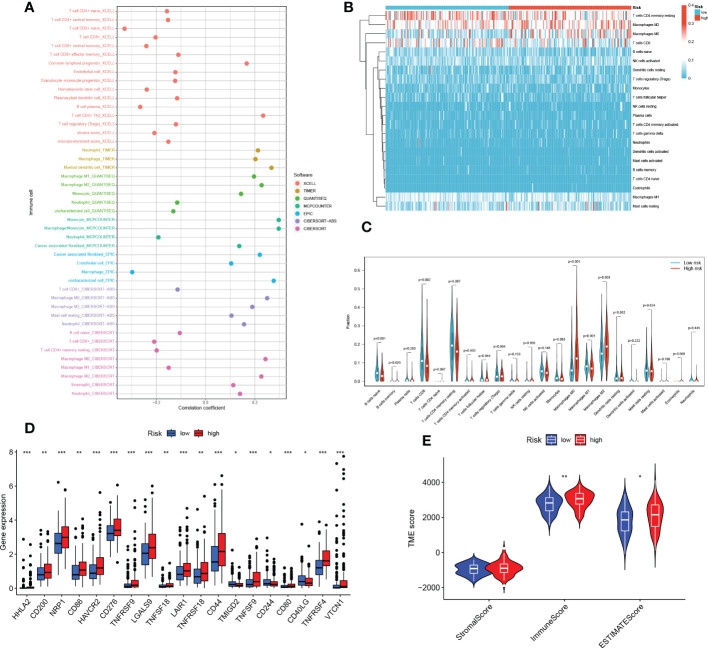
TEX risk score predicts TME and immune cell infiltration. **(A)** The relative proportion of infiltrating immune cells with risk scores. **(B)** The relative proportion of infiltrating immune cells with risk scores. **(C)** Immune cell component between HR group and LR group. **(D)** Immune checkpoint differences between HR and LR groups. **(E)** Estimate the score of the expression profile in the HR group and LR group. *P < 0.05, **P < 0.01, ***P < 0.001.

It has been established that interaction between cancer cells and the TME is crucial for the development and spread of tumors (27). Additionally, TIICs represent a significant part of the TME, and the composition and location of these cells have a direct impact on the formation and occurrence of tumors (31). Therefore, using the algorithms of the XCELL, TIMER, QUANTISEQ, MCPCOUNTER, CIBERSORT, CIBERSORT-ABS, and EPIC platforms, we looked at the relationship between risk scores and tumor immune cells (**Figure 9A**). We quantified the relative proportions of infiltrating immune cells using the CIBERSORT script and then produced a heat map of patients ranked from lowest to highest risk score showing the degree of infiltration corresponding to each immune cell (**Figure 9B**). Moreover, correlations were then analyzed according to HR and LR groups, with a larger proportion of T cells and Macrophages (**Figure 9C**). Due to the significant impact of abnormal expression and function of immune checkpoint molecules on tumor immunotherapy, we analyzed differences in immune checkpoints on the basis of risk scores. In particular, only one immune gene checkpoint, CD40LG, showed upregulation in the LR group, while the rest of the genes showed downregulation in the LR group, including HHLA2, CD200, NRP1, CD86, HAVCR2, CD276, TNFRSF9, LGALS9, TNFSF18, LAIR1 TNFRSF18, CD44, TMIGD2, TNFSF9, CD244, CD80, TNFRSF4, VTCN1 (**Figure 9D**). Thereafter, we used ESTIMATE to calculate the stromal and immune cell proportions of HR and LR groups to estimate tumor purity (**Figure 9E**).

### 3.10 Predicting and validating the efficacy of immunotherapy

**Figure 10:**
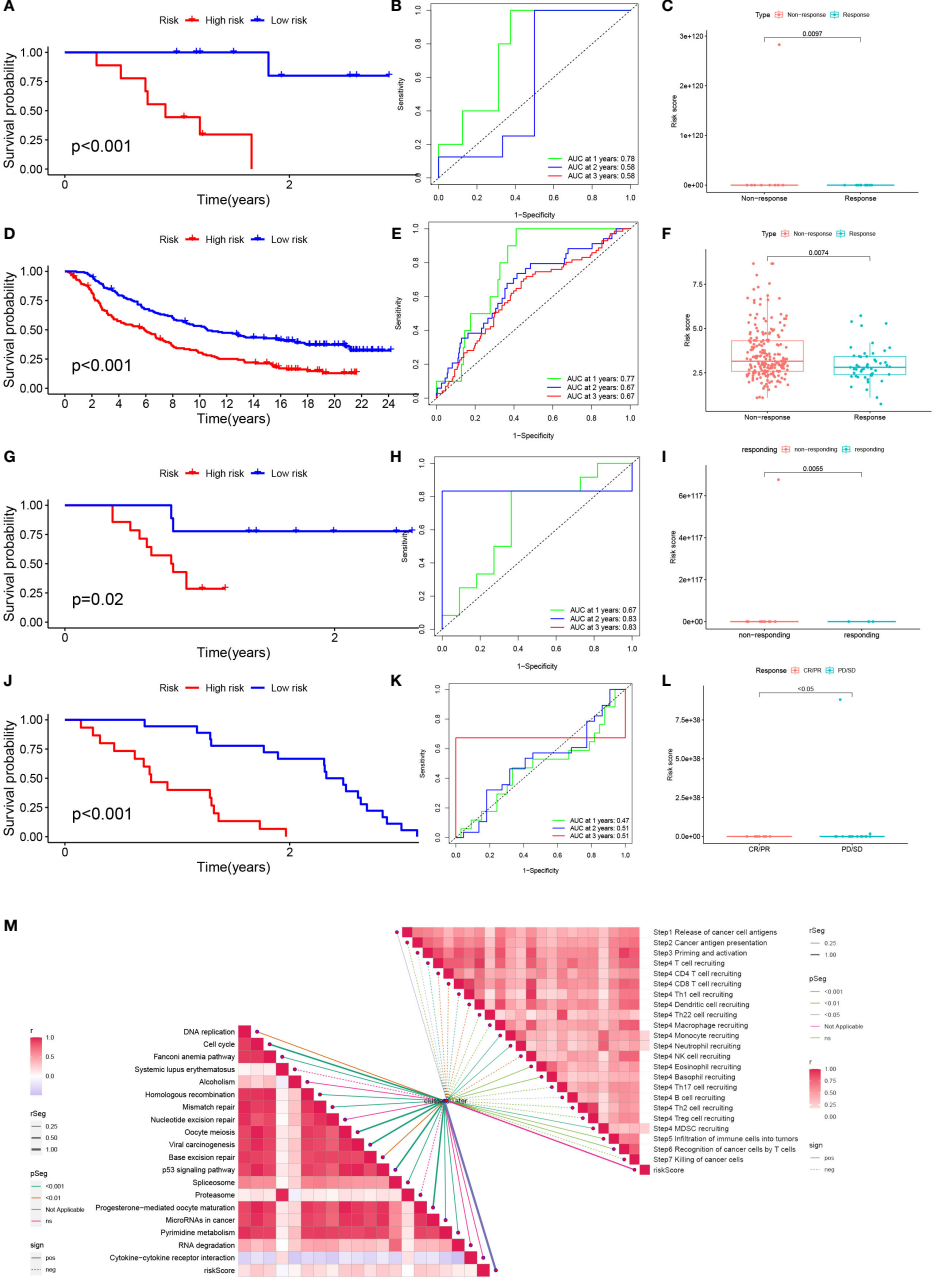
Prediction and validation of immunotherapy effects. **(A)** Survival curves for the HR group and LR group of the GSE78220 cohort. **(B)** Risk score prognostic ROC curves for the GSE78220 cohort. **(C)** Comparison of overall response rates between the HR group and LR group of the GSE78220 cohort. **(D)** Survival curves for the HR group and LR group of the IMvigor cohort. **(E)** Risk score prognostic ROC curves for the IMvigor cohort. **(F)** Comparison of overall response rates between the HR group and LR group of the IMvigor cohort. **(G)** Survival curves of HR group and LR group of GSE79671 cohort. **(H)** Risk score prognostic ROC curves for the GSE79671 cohort. **(I)** Comparison of overall response rates between the HR group and LR group of the GSE79671 cohort. **(J)** Survival curves of HR group and LR group of GSE91061cohort. **(K)** Risk score prognostic ROC curves for the GSE91061 cohort. **(L)** Distribution of risk scores between responders and non-responders in the GSE91061 cohort. **(M)** Correlation of risk scores with ICB response signature and each step of the tumor-immune cycle.

To test the potential of the risk score in predicting immunotherapy from a real immunotherapy cohort, four cohorts of patients receiving immunotherapy were selected. The results showed that patients who responded to immunotherapy had a lower risk score, and the LR group had a higher overall response rate than the HR group (**Figures 10C, F, I, L**). Similarly, in the four cohorts, patients with lower risk may have a better prognosis (**Figures 10A, D, G, J**). Meanwhile, the ROC curve land demonstrated that the TEX signature had a better predictive ability for patient prognosis (**Figures 10B, E, H, K**). ICB is the most well-studied class of immunotherapeutic agents that block inhibitory signals for T-cell activation, enabling tumor-reactive T cells to mount an effective anti-tumor response (29). To further explore the role of risk scores in immunotherapy, we explored the correlation between risk scores and ICB-related positive signals. The results showed that risk score was positively correlated with some signals such as mismatch repair, cell cycle, DNA replication, base excision repair, and viral oncogenic effects (**Figure 10M**). We compared the differences in the activity of the tumor immune steps between the high and LR groups, where a portion of the cycle steps showed upregulated activity, including the release of cancer cell antigens (step 1), NK cell recruitment (step 4), and immune cell infiltration into the tumor. This allowed us to assess the biological functions of the chemokine system and immunomodulators (step 5). Once more, we looked at a stronger negative link between each of these tumor immune cycle phases and risk scores (**Figure 10M**).

### 3.11 Relationship between risk scores and response to chemotherapy

**Figure 11:**
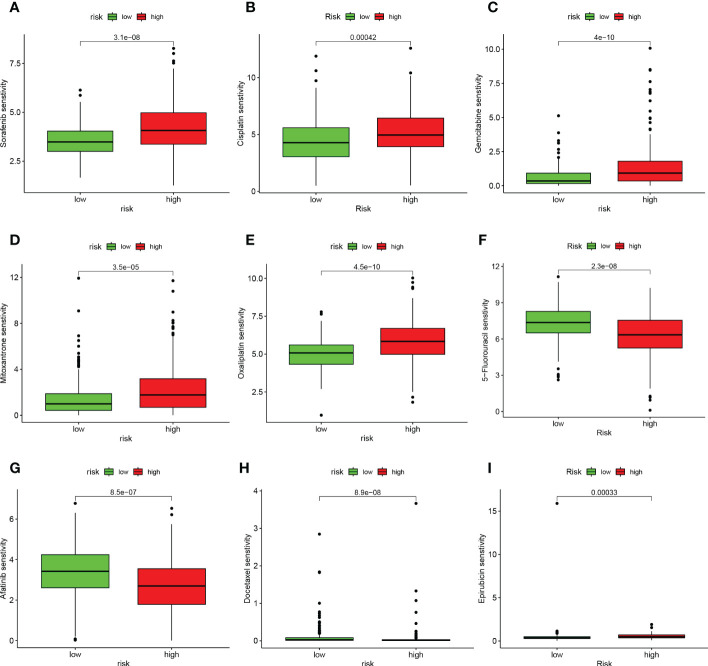
TEX signature characteristics predicted the sensitivity of chemotherapy. **(A)** Sorafenib, **(B)** Cisplatin, **(C)** Gemcitabine, **(D)** Mitoxantrone, **(E)** Oxaliplatin, **(F)** 5-Fluorouracil, **(G)** Afatinib, **(H)** Docetaxel, and **(I)** Epirubicin. Relationship between risk score and ICB response characteristics, and each stage of the tumor immune cycle.

We analyzed the relationship between risk scores and the IC50 values of nine FDA-approved chemotherapies, and immunological agents. As shown in **Figure 11**, Sorafenib, Cisplatin, Gemcitabine, Mitoxantrone, Oxaliplatin, and Epirubicin were found to be more sensitive in the HR group. While patients in the LR group were more sensitive to 5-Fluorouracil, Afatinib, and Docetaxel.

### 3.12 Validation of expression of TEX genes that comprised the risk model by RT-qPCR

**Figure 12:**
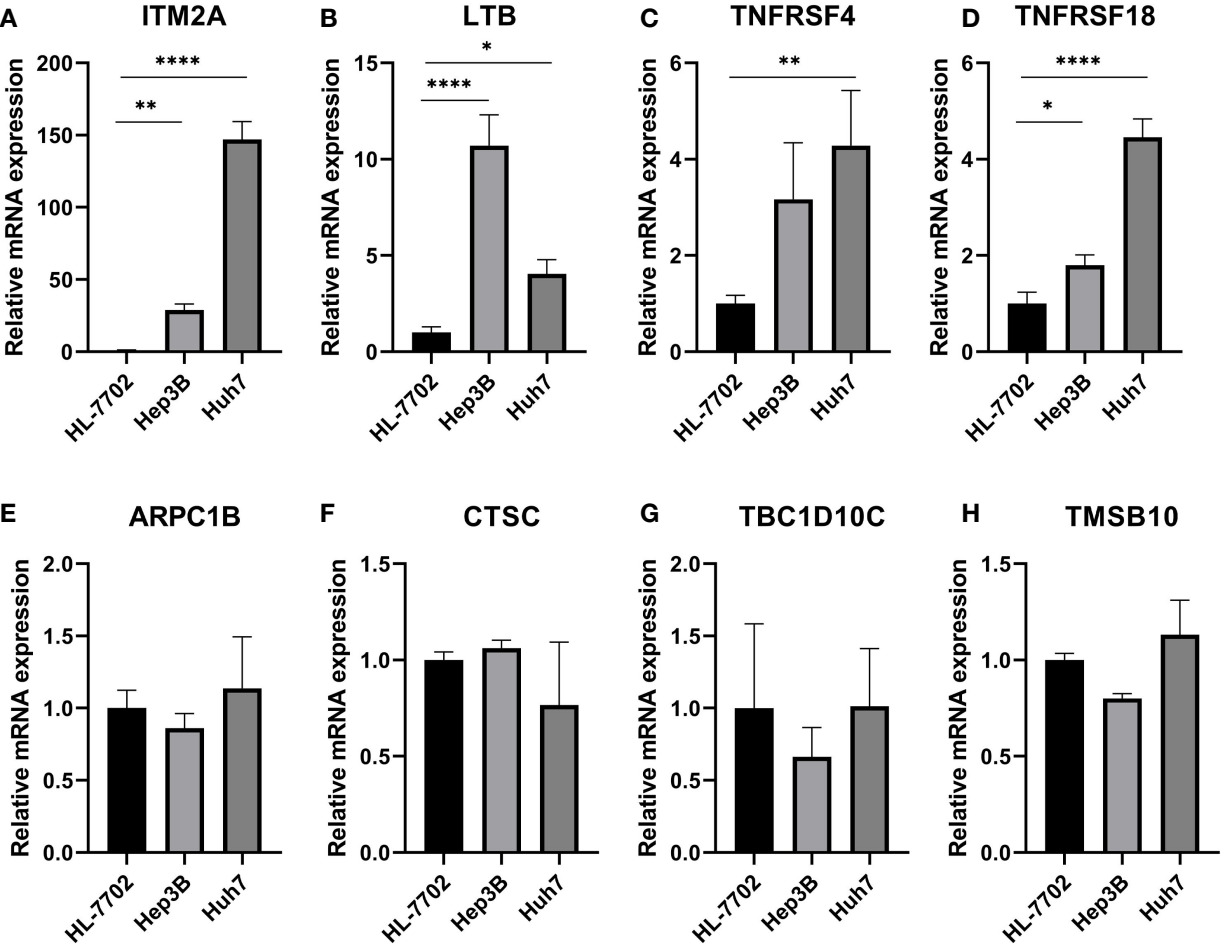
Validation of expression of TEX genes that comprised the risk model by RT-qPCR. QRT-PCR analysis of **(A)** ITM2A, **(B)** LTB, **(C)** TNFRSF4, **(D)** TNFRSF18, **(E)** ARPC1B, **(F)** CTSC, **(G)** TBC1D10C, and **(H)** TMSB10. *P < 0.05, **P < 0.01, ****P < 0.0001.

To verify the expression patterns of TEX-related genes in HCC patients, we performed qRT-PCR analysis. We found that ITM2A (**Figure 12A**), LTB (**Figure 12B**), TNFRSF4 (**Figure 12C**), and TNFRSF18 (**Figure 12D**) were significantly overexpressed in hepatocellular carcinoma cell lines (Hep3B and Huh7) relative to normal liver cell lines (HL-7702). Therefore, we hypothesized that the aberrant expression of these genes likely promoted the malignancy of HCC. However, ARPC1B, CTSC, TBC1D10C, and TMSB10 were not detected to be differentially expressed (**Figures 12E-H**).

## 4 Discussion

A multidimensional comprehensive HCC treatment strategy based on resection, liver transplantation, radiotherapy, chemotherapy, percutaneous ablation, and immunotherapy for early-stage HCC can achieve promising outcomes (32–34). However, due to the low sensitivity of traditional tumor diagnostic methods and the lack of obvious symptoms of early-stage HCC, most patients are in advanced stages of HCC at the time of diagnosis (35, 36). And therapeutic measures to treat advanced HCC are scarce and ineffective (37, 38). Therefore, early diagnosis and treatment as well as the development of new therapeutic measures are of great value to improve the long-term survival of HCC patients. Notably, the treatment response and disease progression of HCC patients vary greatly among individuals due to their different epigenetic statuses, complex tumor microenvironment, and high heterogeneity (39). Traditional tumor staging focuses only on the tumor status at that point in time, and cannot show the dynamically changing tumor microenvironment and immune characteristics, thus failing to accurately predict patient disease progression and treatment response (40). Molecular markers have great potential in this regard (41). T cell exhaustion is defined as a state of dysfunction resulting from persistent exposure of T cells to antigenic and/or inflammatory signals in chronic infections or cancers (6). In this state, dysfunctional T cells, including effector T cells as well as memory T cells, lose the ability to eliminate infection and cancer (42). However, it has been shown that suppressive receptor overexpression is based on T cell exhaustion. Blockades of these receptors such as PD-1 and CTLA-4 can reverse the state and reactivate the immune response thereby stopping tumor progression (6, 42–45), suggesting the great potential of immune checkpoint blockade therapies in this regard. Unfortunately, despite the important value of T cell exhaustion for the development of multiple cancers, including HCC, there are still no systematic studies on T cell exhaustion in HCC. Therefore, we developed a multi-biomarker model based on TEX-related genes that can help physicians assess the prognosis and tumor microenvironment of HCC patients and provide a theoretical basis for individualized and precise treatment.

We obtained T-cell maker genes by dimensionality reduction and clustering based on scRNA-seq data from the GSE166635 dataset. The TCGA-LIHC data and GSVA algorithm were then used to identify the key modules most associated with T cell exhaustion progression. 22 candidate genes for T-cell fatigue were ultimately found when we chose the intersection of T-cell marker genes and module genes. After that, a new prognostic model was created by screening 11 important genes using Lasso regression analysis and multifactorial COX risk regression analysis. A substantial prognostic difference was discovered between the two groups, demonstrating the independent predictive value of the TEX signature we created for HCC. The exceptional predictive efficacy of the TEX signature on patient prognosis was proven by the ROC curve and calibration curve analysis. Additionally, the nomogram we created demonstrates the TEX signature’s superiority to the other clinically used indications in a promising way. Previous studies have shown that LTB, a member of the tumor necrosis factor ligand superfamily, participates in immune cell interactions and regulates cytokine secretion by binding to LT-β receptors and forming heterodimeric complexes with LT-α (46, 47). In HNSCC, LTB binds to EGFR and induces cetuximab resistance 33397394. In addition, it has also been suggested that LTB may mediate NF-κB signaling and thus influence the development of HCV-associated HCC (48). TNFSF4 is overexpressed in HCC and contributes to poor prognosis by activating multiple immunosuppressive pathways (49). Meanwhile, another study found that ivolizumab (TNFRSF4 agonist) is expected to be a new oncologic agent due to its well-tolerated and effective anti-tumor capacity in locally advanced or metastatic HCC (50). In addition, TMSB10 is closely associated with various tumor phenotypes such as cell proliferation, apoptosis, and angiogenesis (51), while overexpressed in HCC tissues and can affect distant tumor metastasis (52). TBC1D10C mediates Map3k3-NF-κB signaling axis activation to inhibit CD8 T cell activation and anti-tumor function thus promoting tumor progression (53). It is considered a tumor immunotherapy target (53). In previous studies, CTSC was considered a key molecule in squamous cell carcinoma as well as breast cancer (54, 55). While another experimental study demonstrated that CTSC promotes HCC cell proliferation and metastasis through activation of the TNF-α/p38 MAPK pathway. Based on the interaction between CTSC and MAPK pathway, it may be useful to predict the sensitivity of HCC patients to Ralimetinib (MAPK inhibitor) for personalized and precise drug therapy.

Mutations in some key genes are critical for tumorigenesis (56, 57). Therefore, we analyzed the mutation probabilities of various genes in HCC samples. The results showed that both TP53 and CTNNB1 had a high mutation probability in the high- and low-risk groups, which is consistent with previous studies (58). Some studies have shown that HCC with CTNNB1 mutation is characterized by high differentiation and better prognosis, but HCC with TP53 mutation and without CTNNB1 mutation is more aggressive and strongly associated with poor prognosis (59). The use of AURKA inhibitors (alisertib) and EZH2 inhibitors (γbogenic acid) in HCC patients with TP53 mutations may result in good outcomes (60). However, it is important to note that tumorigenesis and malignant transformation are often the results of the accumulation of mutations in multiple genes, and a single gene is not sufficient to describe the overall mutational landscape of the tumor (61, 62). TMB refers to the cumulative number of somatic missense mutations and represents the instability of the patient’s genome (63). Generally, high TMB results in more antigenic sites exposed, and the increased antigenic targets have more chances to be recognized by T cells to initiate antitumor effects (64). However, our results suggest that high TMB levels often correspond to a poor prognosis in HCC patients. It has also been shown that HCC usually has lower TMB levels compared to other common tumors (65) and that high TMB is a predictor of poor prognosis in HCC patients after radical hepatectomy (66). However, the exact mechanism of effect remains unclear and more basic studies are needed to explore and demonstrate it.

The important influence of tumor microenvironments in various tumor phenotypes has become a consensus. Immune cell infiltration, as one of the key immune features of the tumor microenvironment, plays a key role in the immune escape of tumor cells and the formation of an inflammatory environment (67). Therefore, we analyzed the differences in the level of immune cell infiltration between the HR and LR groups. It has been shown that naive B cells, and memory B cells are significantly and positively correlated with better survival in HCC patients (68), which is consistent with our findings. Flecken, T. et al. proposed that CD8 T cell recognition of tumor-associated antigens (TAA) to kill tumor cells is a key aspect of the antitumor effect in HCC and that these responses are more robust in early HCC (69). In addition, they also found that CD8 T cells within the tumor failed to produce IFN-γ and exhibited a depleted state, but TAA-specific CD8 T cells in peripheral blood were not affected (69). This may suggest that the antitumor immune response in HCC is still subverted by a suppressive immune microenvironment, further demonstrating the important value of T cell exhaustion for the progression and treatment of HCC. Our results showed that the low-risk group was characterized by high M1 macrophage infiltration while the high-risk group was characterized by high M2 macrophage infiltration. It has been shown that the HCC microenvironment has a greater tendency to induce M2 polarization thereby promoting immunosuppression, angiogenesis, metastasis, and invasion and leading to poor prognosis (70, 71). This tendency may be due to the crosstalk between myeloid-derived suppressor cells (MDSC) and tumor-associated macrophages (TAM) promoting CD4 T cell differentiation into T+H2 phenotype with IL-4 production, which in turn induces the development of M2 macrophages (70). On the one hand, it suggests a better prognosis in the low-risk group, and on the other hand, it demonstrates to some extent the mechanism of the formation of suppressive tumor microenvironment in the high-risk group. The exploration of immune cell infiltration in different risk groups of HCC patients can help clinicians to have a better understanding of the overall immune landscape of patients and the role of immune regulation in the development of tumors.

Immunotherapy based on immune checkpoint inhibitors (ICIs) has become an integral part of various cancer treatment strategies and is being promoted as the first-line treatment for advanced unresectable HCC (72). By assessing immune checkpoint gene expression in different patients, HCC patients who can benefit more from immune checkpoint blockade therapy can be screened for personalized and precise. Some studies show that HHLA2 binds to T cells and inhibits the proliferation of CD4+ and CD8+ T cells and enables immune escape (73). This may be one of the mechanisms of T-cell exhaustion in HCC. In addition, high expression of HHLA2 is significantly positively correlated with high CD8 T-cell infiltration and prognosis of HCC patients and is also considered a potential biomarker for HCC 35084443. It has been demonstrated that CD200 is highly expressed in tumors and surrounding tissues of HCC and can regulate CD4 T-cell expression as well as suppress immune function in HCC patients leading to immune tolerance (74, 75). Mechanistic studies have also shown that NRP1 increases the number of tumors stem cells and mediates EMT-based HCC migration (76). In mouse models of HCC, low expression of CD86 may inhibit the Ag-presenting activity of Kupffer cells (77). In addition, CD276 can mediate the PI3K/AKT/MMPs pathway thereby promoting angiogenic mimic formation in HCC and facilitating HCC growth and metastasis (78). It has been suggested that CD80 not only binds competitively to PD-L1 thereby inhibiting antigen presentation but also binds to CTLA-4 thereby inhibiting T cell responses, both of which together promote immune escape (31, 79). Subsequently, we also validated the accuracy and efficacy of the model in four cohorts of patients receiving immunotherapy, and the results were highly satisfactory. Based on the risk score, clinicians are able to assess the expression of immune checkpoints in patients to develop precise immunotherapy regimens and thus improve outcomes.

Although the TEX signature we constructed is outstanding in its ability to identify the immune landscape of patients and to predict their prognosis. Some limitations, however, still require us to acknowledge and find appropriate ways to address them in subsequent studies. First, the TCGA-LIHC dataset we included was predominantly white and more data from other ethnicities need to be collected for validation subsequently. Data analysis is based on public database data, which may lead to deviations in prediction results from the actual situation. Although we have taken several approaches to try to avoid this situation, more data from HCC patients need to be collected to validate the utility of the model and the accuracy of the prediction of immunotherapy. In addition, more prospective studies as well as mechanistic studies are needed to refine the details related to this study.

## 5 Conclusion

TEX signature is a novel predictive biomarker and a possible therapeutic target for patients with HCC, as we have shown for the first time. Additionally, the TEX signature can characterize the immunological milieu of HCC patients and appropriately estimate the prognosis of HCC patients, which can assist doctors in identifying certain patient subgroups that may benefit from immunotherapy and chemotherapy for individualized treatment.

## Data availability statement

The datasets presented in this study can be found in online repositories. The names of the repository/repositories and accession number(s) can be found in the article/**Supplementary Material**.

## Author contributions

HC and JQZ conceived the study. HC, SZ, JY, XG, GP, JZ, XX, and GS drafted the manuscript. HC and KX performed the literature search and collected the data. HC, SZ, and JY analyzed and visualized the data. XG and HC completed *in vitro* experiments. ZX, SC and JQZ helped with the final revision of this manuscript. All authors contributed to the article and approved the submitted version.
