# Supplementary material for: T-cell exhaustion signatures characterize the immune landscape and predict HCC prognosis via integrating single-cell RNA-seq and bulk RNA-sequencing
Source: Front Immunol. 2023 Mar 15;14:1137025. doi: 10.3389/fimmu.2023.1137025 (PMC10050519; doi:10.3389/fimmu.2023.1137025)
Supplement: Supplementary file 3 [file Presentation_1.pdf]

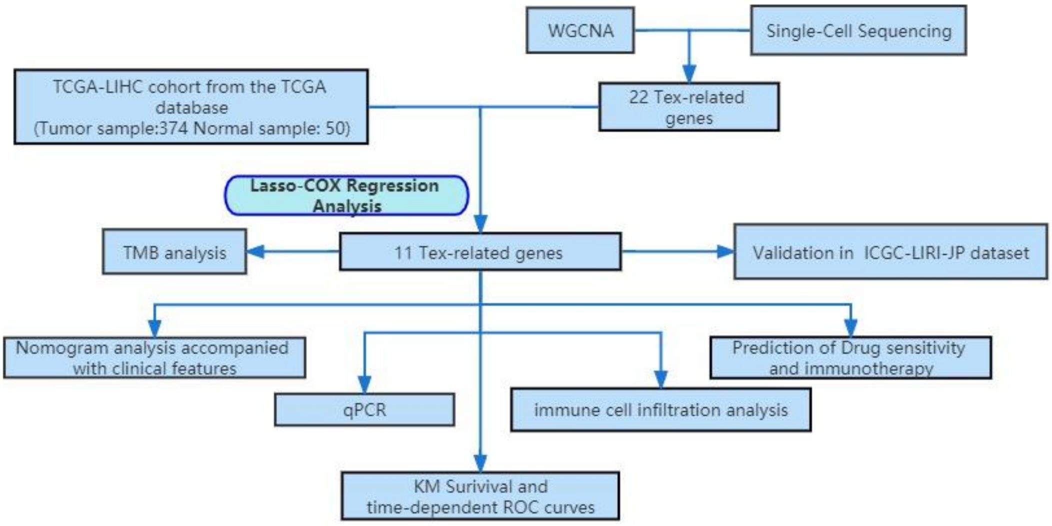

Supplementary Figure 1 The flowchart summarizes the main design of the present study.

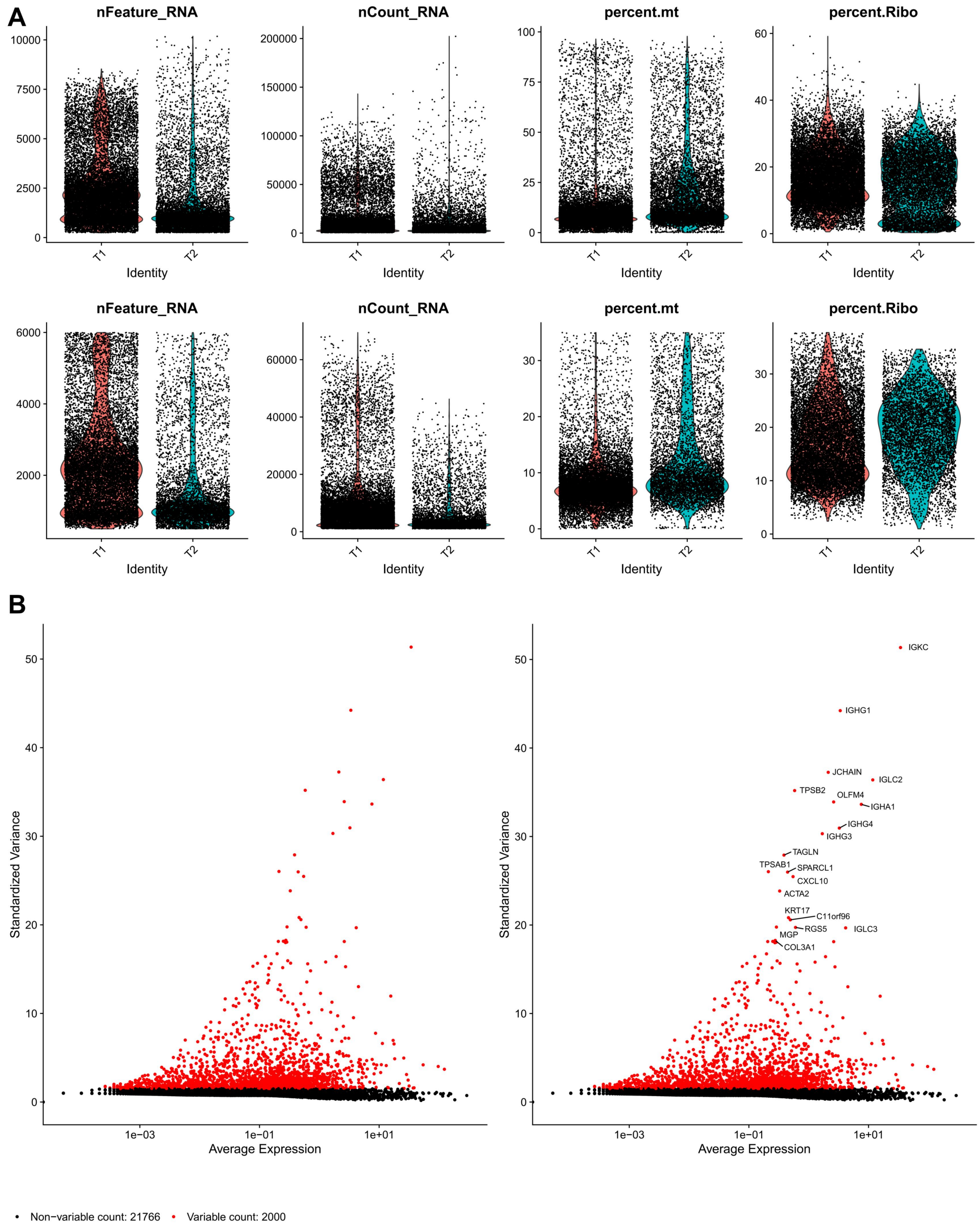

Supplementary Figure 2 Quality Control. (**A**) After cell quality control (QC),18986 cells were identified. (**B**) Top 2000 highly variable genes.

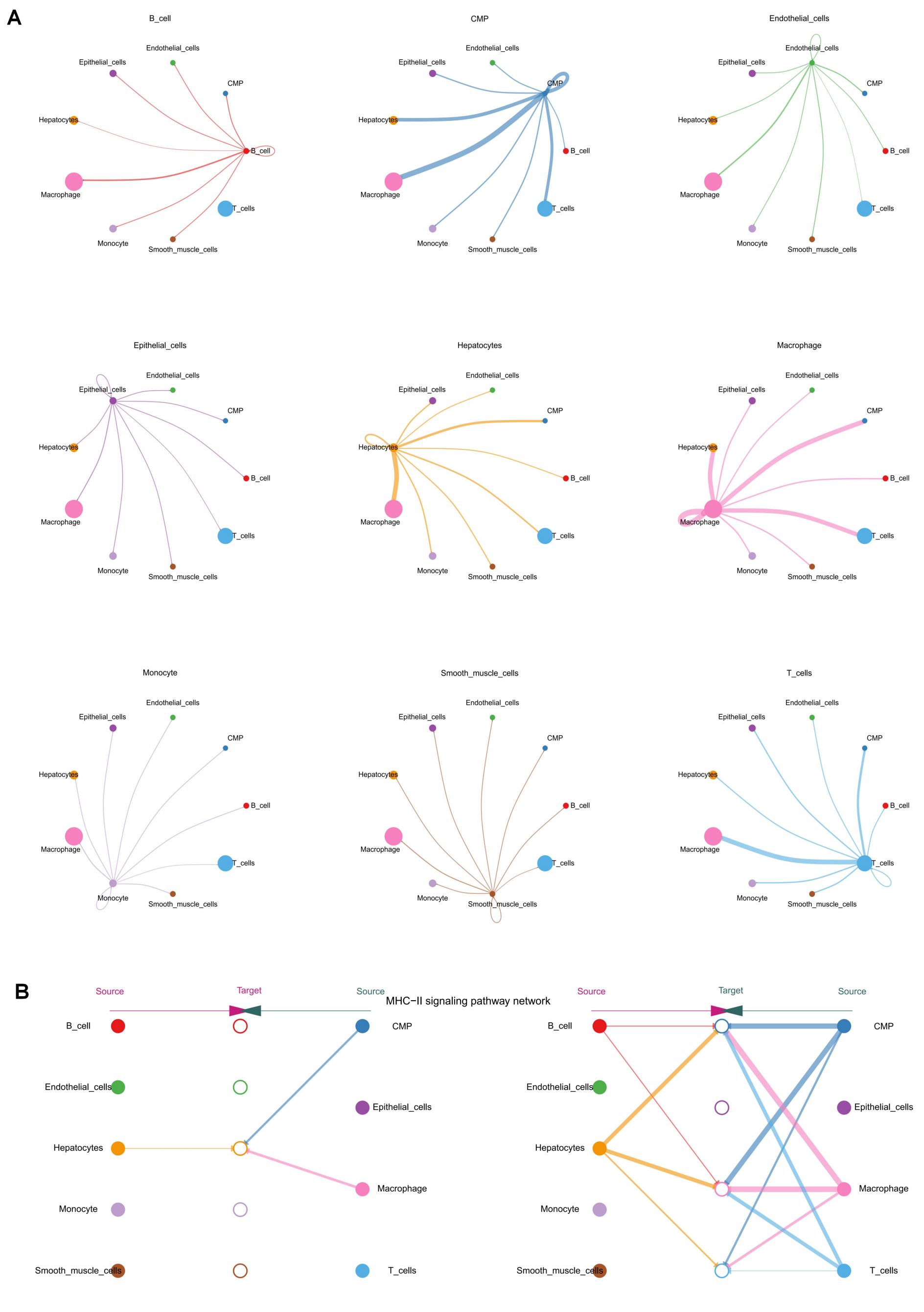

Supplementary Figure 3 Cell Communication Analysis. (**A**)Cellular communication network of ligand-receptors between each cell subpopulation and other cells. (**B**) Communication networks between the MHC-II signaling pathway and other cellular subpopulations
